# Effects of *Fortunella margarita* Fruit Extract on Metabolic Disorders in High-Fat Diet-Induced Obese C57BL/6 Mice

**DOI:** 10.1371/journal.pone.0093510

**Published:** 2014-04-04

**Authors:** Si Tan, Mingxia Li, Xiaobo Ding, Shengjie Fan, Lu Guo, Ming Gu, Yu Zhang, Li Feng, Dong Jiang, Yiming Li, Wanpeng Xi, Cheng Huang, Zhiqin Zhou

**Affiliations:** 1 College of Horticulture and Landscape Architecture, Southwest University, Chongqing, China; 2 Drug Discovery Lab, School of Pharmacy, Shanghai University of Traditional Chinese Medicine, Shanghai, China; 3 Key Laboratory of Horticulture Science for Southern Mountainous Regions, Ministry of Education, Chongqing, China; 4 Citrus Research Institute, Chinese Academy of Agricultural Sciences, Chongqing, China; Consiglio Nazionale delle Ricerche, Italy

## Abstract

**Introduction:**

Obesity is a nutritional disorder associated with many health problems such as dyslipidemia, type 2 diabetes and cardiovascular diseases. In the present study, we investigated the anti-metabolic disorder effects of kumquat (*Fortunella margarita* Swingle) fruit extract (FME) on high-fat diet-induced C57BL/6 obese mice.

**Methods:**

The kumquat fruit was extracted with ethanol and the main flavonoids of this extract were analyzed by HPLC. For the preventive experiment, female C57BL/6 mice were fed with a normal diet (Chow), high-fat diet (HF), and high-fat diet with 1% (w/w) extract of kumquat (HF+FME) for 8 weeks. For the therapeutic experiment, female C57BL/6 mice were fed with high-fat diet for 3 months to induce obesity. Then the obese mice were divided into two groups randomly, and fed with HF or HF+FME for another 2 weeks. Body weight and daily food intake amounts were recorded. Fasting blood glucose, glucose tolerance test, insulin tolerance test, serum and liver lipid levels were assayed and the white adipose tissues were imaged. The gene expression in mice liver and brown adipose tissues were analyzed with a quantitative PCR assay.

**Results:**

In the preventive treatment, FME controlled the body weight gain and the size of white adipocytes, lowered the fasting blood glucose, serum total cholesterol (TC), serum low density lipoprotein cholesterol (LDL-c) levels as well as liver lipid contents in high-fat diet-fed C57BL/6 mice. In the therapeutic treatment, FME decreased the serum triglyceride (TG), serum TC, serum LDL-c, fasting blood glucose levels and liver lipid contents, improved glucose tolerance and insulin tolerance. Compared with the HF group, FME significantly increased the mRNA expression of PPARα and its target genes.

**Conclusion:**

Our study suggests that FME may be a potential dietary supplement for preventing and ameliorating the obesity and obesity-related metabolic disturbances.

## Introduction

Obesity has increasingly become a major health problem all over the world [1], and it is predicted to affect more than one billion people by the year 2020 [2]. The primary co-morbidity of obesity is metabolic syndrome, which is characterized by hypertension, dyslipidemia, hyperglycemia along with insulin resistance [3], [4]. The commonly used therapy for glucose and lipid metabolism disorder is drug intervention, which is costly and low efficacy [5]. In addition, the pharmacological approaches for obesity treatment were associated with many side effects such as liver toxicity [6]. Therefore, in recent years much attention has been paid to dietary prevention and intervention for obesity and obesity- related disorders [7]–[10].

Citrus fruits are widely consumed all over the world and their peels, the most useful part of the fruits in terms of bioactive compounds were discarded because of bad flavor [11]–[13]. The existing studies have suggested that citrus peels played important roles in regulating glucose and lipid metabolic disorders. Kang et al. reported that *Citru*s *sunki* peel extract reduced the body weight, adipose tissue weight gain and inhibited the accumulation of fatty droplets in high-fat diet-induced obese mice [14]. Ding et al. have also reported that *Citrus ichangensis* peel extract could ameliorate metabolic disorders [13]. Citrange fruit extracts were able to alleviate obesity-associated metabolic disorders as the latest research reported [15]. *Fortunella margarita* Swingle (FM) belongs to the genus *Fortunella* Swingle, a close relative of the genus *Citrus* L, and both genera were grouped into the true citrus group of the family of Rutaceae. FM fruits, when eaten fresh, has a sweet outer peel and a tart, juicy flesh [16]. FM fruits can also be processed into jam, sauce, marmalade, liqueur and other products. Moreover, in China, FM has been traditionally used as a kind of folk medicine to treat cold, cough and inflammatory diseases for centuries [17]. It was also found that FM fruits have high radical scavenging capacities and strong antioxidant activity, and consumption of FM fruits may be of health-promotion [16].

There is an increasing evidence that the control of obesity and obesity-related metabolic diseases is closely related to the regulation of peroxisome proliferator-activated receptors (PPARs) signaling [18]. PPARs, the ligand-activated nuclear transcription factors consist of three members named PPARα, PPARβ/δ and PPARγ, which regulate the expression of lipogenic and glucose metabolic genes. Among PPARs, PPARα is highly expressed in liver and regulates the expression of genes involved in lipid catabolism [18], [19]. As reported in current literature, many natural compounds have been suggested to regulate metabolic disorders via the modulation of PPARs expression [20], [21]. Especially, there are increasing evidences that citrus compounds can regulate PPARs and its target genes transcription, and then modulate the glucose and lipid metabolism of obese mice [22]–[24]. Naringenin, for example, could increase hepatic fatty acid oxidation through co-activating PPARα and PPARγ transcription, and then prevent lipogenesis in liver, improve glucose tolerance and insulin sensitivity in *Ldlr^−/−^* mice [25]. Nobiletin has also been reported to improve obesity and insulin resistance via increasing PPARγ expression in high-fat diet-induced obese mice [26].

Recently, it has been found that FM is a rich resource of bioactive polyphenols including phenolic acids, flavones, flavanones, flavonols and chalcones, and the bioactive compounds in FM are much different from those of *Citrus* species [27], [28]. In the present study, we investigated whether a long-term administration of *Fortunella margarita* fruit extract (FME) would have beneficial effects on the prevention and treatment of obesity and its related metabolic diseases. The body weight gain, lipid accumulation and gene expression involved in glucose and lipid metabolism in HF diet-induced C57BL/6 mice were investigated.

## Materials and Methods

### Reagents

Ethanol, acetic acid and chloroform were obtained from Sinopharm Chemical Reagent Co., Ltd (Beijing, China). Acetonitrile (HPLC grade) was purchased from J&K Scientific Ltd. (Beijing, China). The standards (purity >98%) were purchased from Shanghai R&D Center for Standardization of Chinese Medicines (Shanghai, China). Unless otherwise noted, all other chemicals were purchased from Sigma Chemical Co. (St. Louis, MO).

### Preparation of FME

FM fruits at commercial maturity stage were collected from the National Citrus Germplasm Repository, Citrus Research Institute of Chinese Academy of Agricultural Sciences, Chongqing, China. The fruits were extracted by 95% ethanol with a solid to liquid ratio of 1∶4 at 85°C for 2 h. The extract was cooled and filtered and the filtered solution was concentrated at 40°C with a rotary evaporator under reduced pressure, freeze-dried to a powder. The powder was stored at −80°C until use. The frozen dried powder of FME was added to the HF diet for experiment.

### HPLC Analysis

The total polyphenols and flavonoids of FME were determined as previously described [29]. The flavonoid composition was determined by the method described previously [13]. Briefly, flavonoids were analyzed with an Agilent 1200 liquid chromatographic system (Agilent Technologies, Santa Clara, CA, USA) with a C18 HPLC Column (250 mm × 4.6 mm, 5 µ, Agilent Technologies, Santa Clara, CA, USA). The mobile phase consisted of 100% acetonitrile (A) and water containing 0.5% acetic acid (B) at a flow rate of 1.0 mLmin^-1^. The UV diode array detector was set at 280 nm and sample injection volume was 10 μl.

### Animals and Diets

All the animal study protocols were approved by the Insitutional Animal Care and Use Committee of Shanghai University of Traditional Chinese Medicine (Approved Number: 2013007). 10–12 weeks old female C57BL/6 mice were purchased from the SLAC Laboratory (Shanghai, China). Mice were maintained under 22–23°C on 12 h light/dark cycle, food and water provided ad libitum.

After a one-week adaptation period, the mice were randomly divided into three groups (n = 7) based on their body weights. For preventive study, mice were fed with a chow diet (10% of calories derived from fat, New Brunswick, NJ, USA, Research Diets; D12450B), or an HF diet (60% of calories derived from fat, New Brunswick, NJ, USA, Research Diets; D12492), or an HF diet supplemented with 1% FME (HF+FME) for 8 weeks. Body weight and food intake were measured every other day. In the therapeutic study, the female C57BL/6 mice were fed a high-fat diet for 3 months to induce obesity. The obese mice were divided into two groups randomly, and then fed with HF or HF+FME diet for another 2 weeks, respectively, with another seven normal mice fed chow diet as control group. Food intake and body weight were recorded every other day.

### Intraperitoneal Glucose and Insulin Tolerance Tests

For glucose tolerance test, the C57BL/6 mice were fasted for 12 h and a basal blood glucose levels (0 min) were measured from the tail vein. Then the mice were intraperitoneally injected with glucose (1 g/kg body weight) and additional blood glucose levels were measured at 15, 30, 60 and 90 min.

For insulin tolerance test, mice were intraperitoneally injected with 0.75 U/kg insulin (Cat No: I 9278, Sigma, St. Louis, MO, USA) without fasting. Before the injection of insulin, the blood samples were collected from the tail vein for measurement of basal blood glucose levels (0 min), and additional blood glucose levels were measured at 15, 30, 60 and 90 min.

### Serum Chemistry Analysis

After overnight fasting (12 h), all mice were anesthetized with urethane before collecting blood samples for analysis. Blood samples were drawn from the heart into a vacuum tube, and serum samples were separated from the blood. Serum triglyceride (TG), total cholesterol (TC), high-density lipoprotein cholesterol (HDL-c) and low-density lipoprotein cholesterol (LDL-c) levels were measured using a Hitachi 7020 Automatic Analyzer (Hitachi, Tokyo, Japan).

### Liver Lipid Content Analysis

The liver and other tissues were collected rapidly at the end of treatment, frozen in liquid nitrogen, and stored at −80°C for the following experiments. To test the liver lipid content, 50 mg of frozen liver tissues were homogenized in 1 ml lysis buffer (20 mM Tris-HCl, pH 7.5, 150 mM NaCl, 1% Triton) and mixed with an equal volume of chloroform. The chloroform layer was separated, dried, and resuspended in isopropyl alcohol to measure the lipid levels as described by Zang [30].

### Morphological Analysis of Epididymal WAT

Frozen white adipose tissue (WAT) samples were fixed in 10% formaldehyde and paraffin-embedded. The sections were stained with hematoxylin and eosin and examined under the light microscope (×200) using the Olympus image analysis software system (Olympus America, Melville, NY, USA). Their surface areas were acquired with Olympus CellSens Standard software (Version 1.5).

### Quantitative Real-time PCR

The quantitative real-time PCRs were performed as described by Fan [31]. Briefly, the total RNA from liver and brown adipocyte tissue (BAT) was extracted with Trizol (Takara, Tokyo, Japan). The first-strand cDNA was synthesized with a cDNA synthesis kit (Fermentas, Madison, WI, USA). The gene expression levels were analyzed using an ABI StepOnePlus Real-Time PCR system (Applied Biosystems, Carlsbad, CA, USA). The primers (Generay Biotech Co., Ltd, Shanghai, China) used in the experiment were shown in Table 1. All results were obtained from at least three independent experiments. β-actin was used as an internal control to normalize the mRNA levels of all genes.

**Table 1:** Sequences of the primers used in real-time PCR.

Gene	Forward primer	Reverse primer
β-Actin	TGTCCACCTTCCAGCAGATGT	AGCTCAGTAACAGTCCGCCTAGA
PPARα	AGGCTGTAAGGGCTTCTTTCG	GGCATTTGTTCCGGTTCTTC
PPARγ	CGCTGATGCACTGCCTATGA	AGAGGTCCACAGAGCTGATTCC
CD36	GCTTGCAACTGTCAGCACAT	GCCTTGCTGTAGCCAAGAAC
LPL	ATCGGAGAACTGCTCATGATGA	CGGATCCTCTCGATGACGAA
ACC	GAATCTCCTGGTGACAATGCTTATT	GGTCTTGCTGAGTTGGGTTAGCT
ABCA1	GGCAATGAGTGTGCCAGAGTTA	TAGTCACATGTGGCACCGTTTT
CYP4a10	GAGTGTCTCTGCTCTAAGCCCA	AGGCTGGGGTTAGCATCCTCCT
CYP4a14	TGAATTGCTGCCAGATCCCACCAGGATC	GTTCAGTGGCTGGTCAGA
ACO	CAGCACTGGTCTCCGTCATG	CTCCGGACTACCATCCAAGATG
UCP1	CATCACCACCCTGGCAAAA	AGCTGATTTGCCTCTGAATGC
UCP2	GGGCACTGCAAGCATGTGTA	TCAGATTCCTGGGCAAGTCACT
UCP3	TGGCCCAACATCACAAGAAA	TCCAGCAACTTCTCCTTGATGA
SCD-1	TCACCTTGAGAGAAGAATTAGCA	TTCCCATTCCCTTCACTCTGA

### Statistical Analysis

All values were expressed as mean ± SEM. Data were analyzed by the SPSS package (Version 16.0, SPSS, Chicago, IL, USA). One-way ANOVA model and Duncan’s multiple range tests were used to determine the significance of differences between groups (*p*<0.05). Origin Pro 8.0 SR4 (Origin Lab, Northampton, MA, USA) were used to make graphs.

## Results

### Polyphenols and Flavonoids in FME

**Figure 1:**
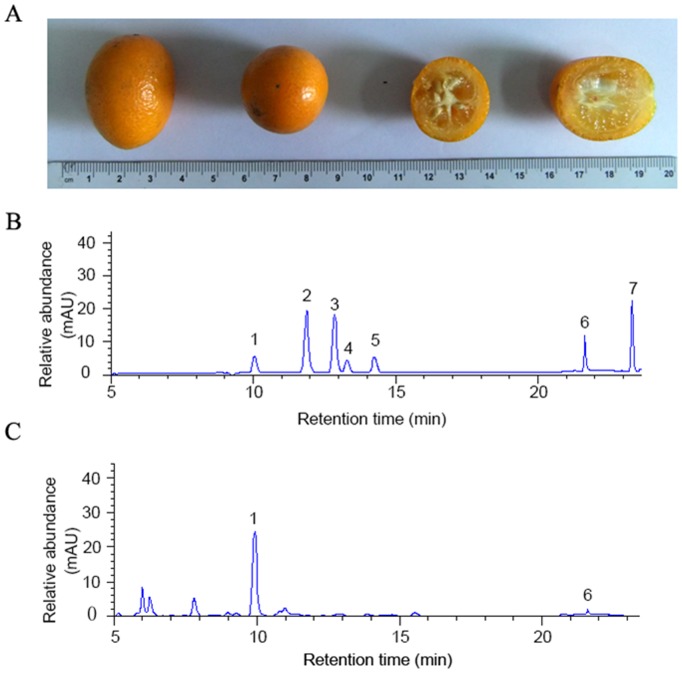
Fruit picture and HPLC chromatograms of major flavonoids of FME. (A) Fruit picture. (B) Chromatograms of standards: (1) neoeriocitrin; (2) narirutin; (3) naringin; (4) hesperidin; (5) neohesperidin; (6) poncirin; (7) naringenin. (C) The major flavonoids of FME were determined by comparing with the chromatogram of standards. (1) neoeriocitrin; (6) poncirin.

The total polyphenols and flavonoids of FME were determined and their contents were 21.94 mg/g and 6.02 mg/g, respectively. The flavonoid composition of FME was analyzed by HPLC method and the results were shown in Fig. 1. Compared with the flavonoids standards of citrus fruit (Fig. 1B), only neoeriocitrin and poncirin were detected in FME (Fig. 1C).

### FME Blocked the High-fat Diet-induced Obesity and Hyperglycemia in C57BL/6 Mice in the Preventive Experiment

**Figure 2:**
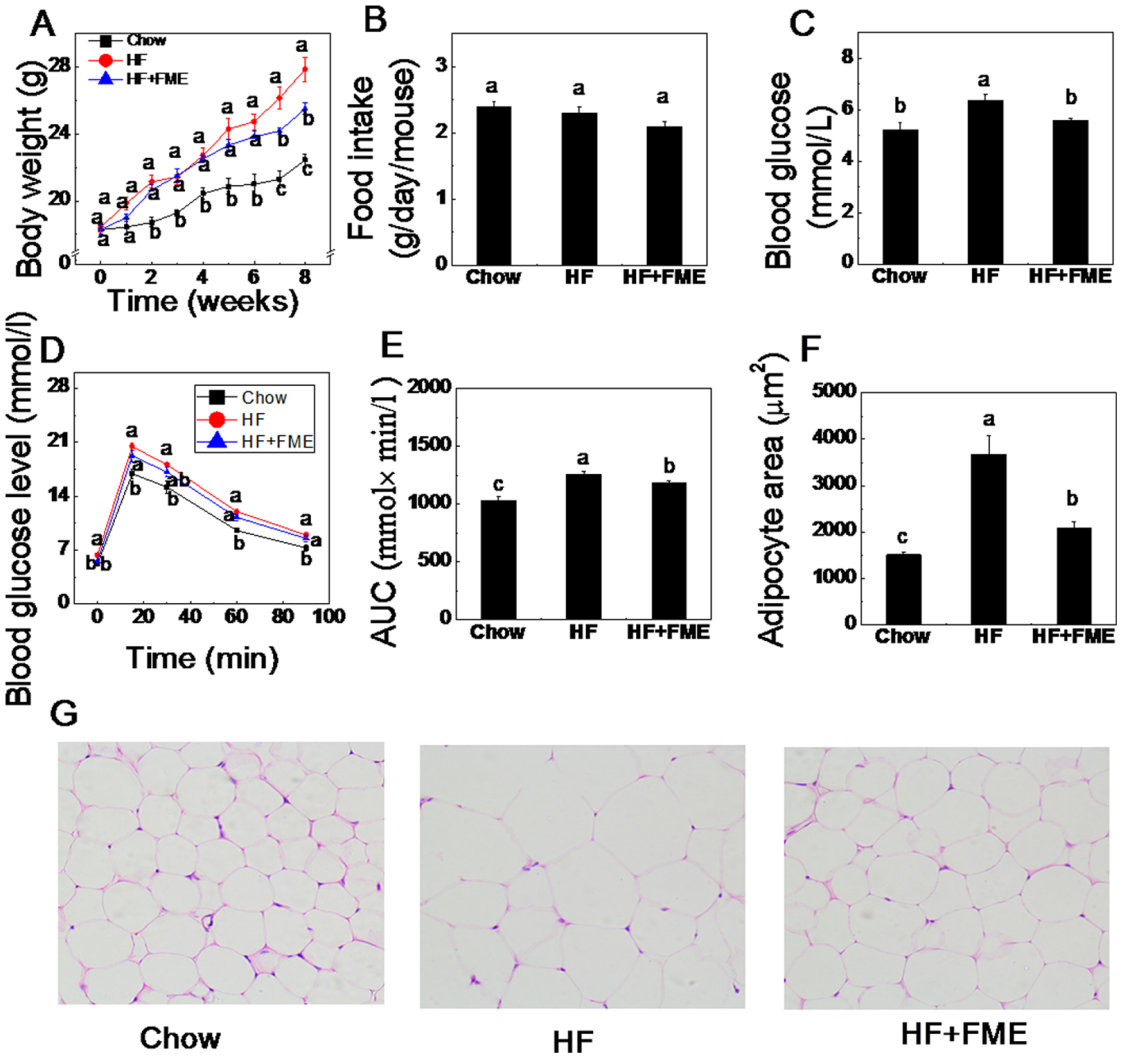
FME blocked the high-fat diet-fed obesity and hyperglycemia in C57BL/6 mice in the preventive treatment. (A): Body weight change. (B): Food intake amount. (C): Fasting blood glucose levels. (D): Intraperitoneal glucose tolerance test (GTT). (E): The area under the curve (AUC) during GTT. (F): White adipocyte areas. (G): H&E staining (×200) of WAT. Values are presented as mean ± SEM (n = 7). Values with different letters were significantly different (*p*<0.05).

To test whether FME prevents high-fat diet-induced body weight gain and metabolic disorders, C57BL/6 mice were fed a standard diet (Chow), a high-fat diet (HF) alone or supplemented with 1% FME (HF+FME) diet for 8 weeks. As shown in Fig. 2A, the body weight of the mice fed with a high-fat diet was significantly higher than those of chow diet-fed mice, indicating the HF diet-induced obesity. The body weight gain induced by high-fat diet was obviously blocked by the supplement of FME (HF+FME group). In this study, there was no significant difference in food intake in terms of weight among the three groups (Fig. 2B). The average caloric intakes in Chow, HF and HF+FME groups were 9.21 Kcal, 12.08 Kcal, 11.22 Kcal respectively per day per mouse. This result indicated that there is no significant difference of energy intake in HF and HF+FME group and showed that the body weight decrease in FME treated mice was not result from the lower caloric intake.

To test the effect of FME on hyperglycemia in high-fat diet-fed mice, fasting blood glucose levels and glucose tolerance test (GTT) were measured. Fig. 2C showed that high-fat diet significantly increased the fasting blood glucose levels in the HF group compared to the Chow group mice (*p*<0.01). Interestingly, the HF+FME group showed a significant decrease in fasting blood glucose levels compared to the HF group (*p*<0.01). During the GTT, the glucose levels in HF+FME group mice were not significantly decreased except the one at 0 min, but the AUC in HF+FME was significant lower than that in the HF group (Fig. 2D, E). Our data suggest that FME effectively prevents the development of hyperglycemia in high-fat diet-fed mice.

We also analyzed the cell size of white adipocytes in high-fat diet-fed mice. As shown in Fig. 2F, G, the size of white adipocytes was significantly increased in the HF group compared to the Chow group after 8 weeks, and FME treatment reduced the size of white adipocytes of HF+FME group compared with high-fat diet-fed mice. These data suggest that FME may prevent the high-fat induced increase in fat mass.

### FME Decreased Serum Lipid Levels in High-fat Diet-fed Mice in the Preventive Experiment

**Figure 3:**
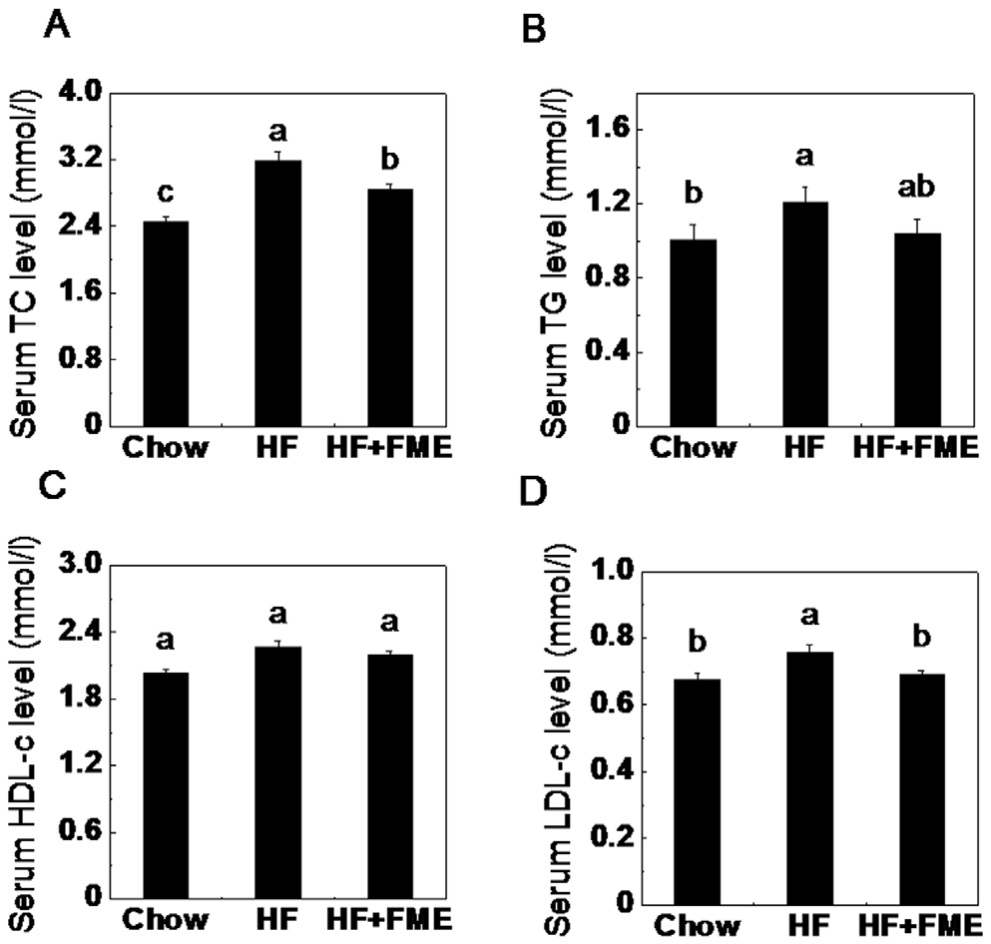
FME decreases serum lipid levels in C57BL/6 mice in the preventive treatment. (A): Total cholesterol. (B): Serum triglyceride. (C): High-density lipoprotein cholesterol. (D): Low-density lipoprotein cholesterol. Values are presented as mean ± SEM (n = 7). Values with different letters were significantly different (*p*<0.05).

To examine the dyslipidemia-preventing effect of FME in high-fat diet-fed mice, the serum lipid levels were analyzed. As shown in Fig. 3A, B and D, the fasting serum TC, TG and LDL-c levels of HF group were significantly increased compared to those of Chow group. FME treatment significantly decreased the serum TC and LDL-c levels of the HF+FME group (Fig. 3A, D). In addition, a lower TG levels in the HF+FME groups were also observed, but no significant difference in HDL-c levels among the three groups was found. These results suggest that FME may be useful in preventing high-fat diet-induced risk of dyslipidemia.

### FME Improves Hyperglycemia in Obese C57BL/6 Mice in the Therapeutic Experiment

**Figure 4:**
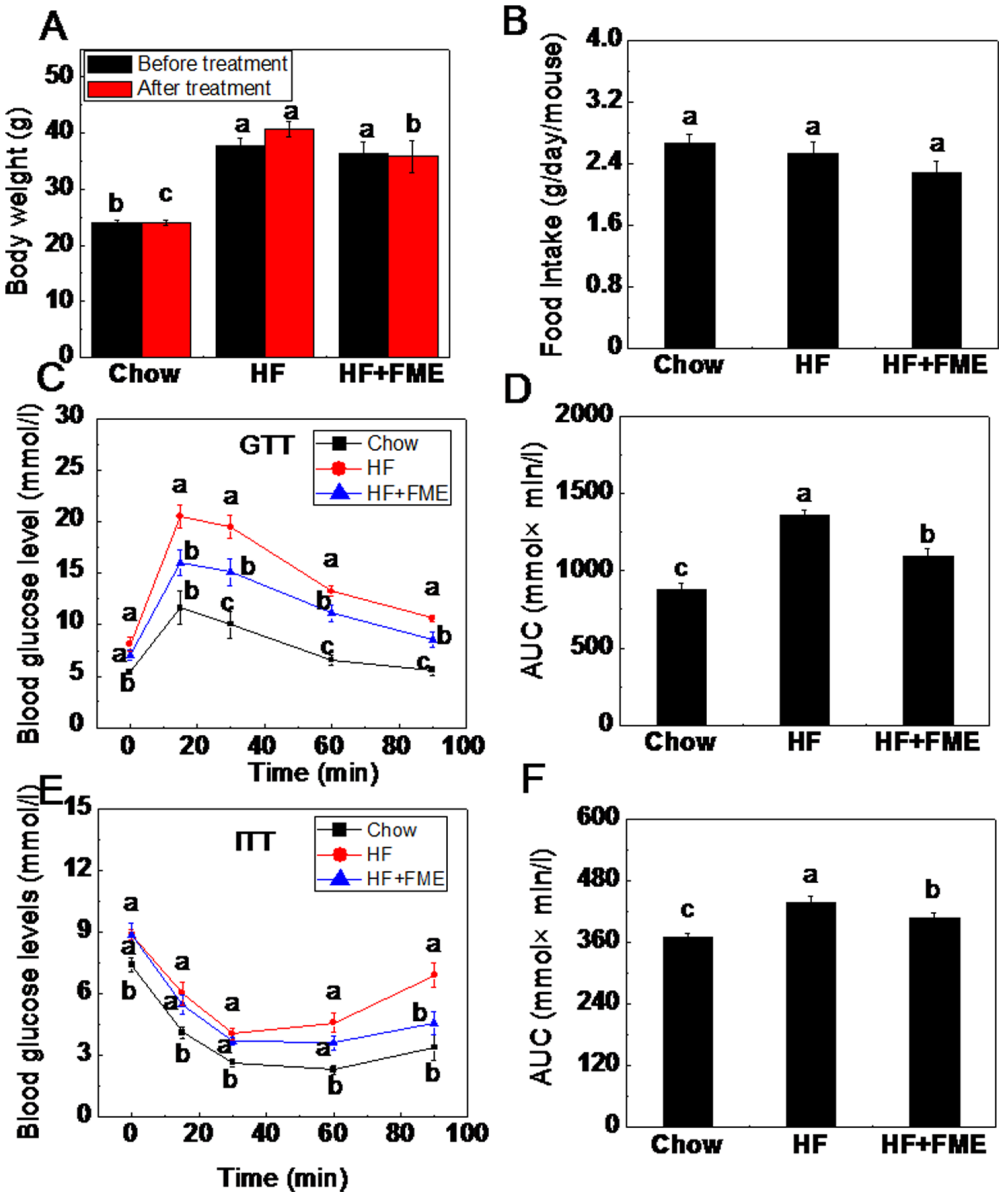
FME improves hyperglycemia in obese C57 BL/6 mice in therapeutic treatment. (A) Body weights before and after treatment. (B) Food intake amount. (C) Intraperitoneal glucose tolerance test (GTT). (D) The area under the curve (AUC) during GTT. (E) Intraperitoneal insulin tolerance test (ITT). (F) The area under the curve (AUC) during ITT. Values are expressed as means ± SEM (n = 7). Values with different letters were significantly different (*p*<0.05).

To evaluate the treatment effect of FME, C57BL/6 mice were fed with a high-fat diet for 3 months to induce obesity. Then, the obese mice were divided into two groups randomly and fed with HF diet alone or HF supplemented with 1% FME for another two weeks. As shown in Fig. 4A, the body weight in HF group mice were increased by 11.7% during the two weeks treatment. However, the body weight in HF+FME group mice maintains the same level before and after the FME treatment. During the treatment, the food intake as weight was similar among the three groups (Fig. 4B) and the average energy intake of mice in Chow, HF and HF+FME groups were 10.24 Kcal, 13.26 Kcal, 12.02 Kcal respectively per day per mouse. This result indicated that there is no significant difference of energy intake in HF and HF+FME group.

To further study whether FME could alleviate the hyperglycemia, GTT and ITT were measured in obese mice. As shown in Fig. 4C, D, the mice with high-fat diet induced obesity displayed impaired glucose tolerance compared with the Chow group mice. FME supplement significantly improved the impaired glucose tolerance at 15, 30, 60 and 90 min (*p*<0.05), indicating that FME may improve glucose tolerance in obese mice. Moreover, the obese mice induced by high-fat diet exhibited impaired insulin tolerance compared with the Chow group mice, whereas, FME treatment significantly decreased the blood glucose levels at 90 min following intraperitoneal injection of insulin (Fig. 4E, F). Taken together, FME significantly attenuated hyperglycemia in the obese mice.

### FME Improves Lipid Profile in Obese C57BL/6 Mice in the Therapeutic Experiment

**Figure 5:**
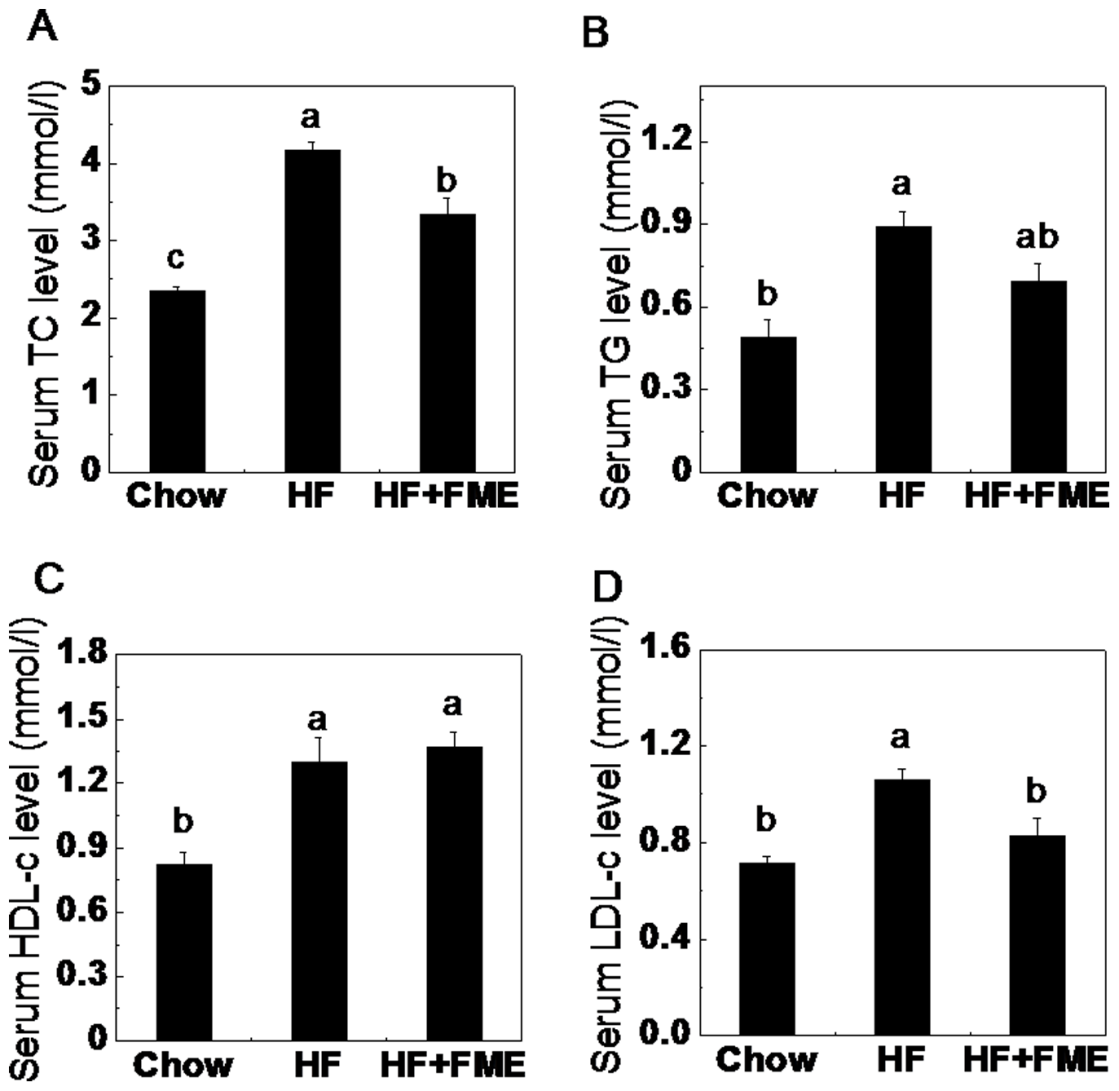
FME improves lipid profile in obese C57 BL/6 mice in the therapeutic treatment. (A): Total cholesterol. (B): Serum triglyceride. (C): High-density lipoprotein cholesterol. (D): Low-density lipoprotein cholesterol. Values are presented as mean ± SEM (n = 7). Values with different letters were significantly different (*p*<0.05).

Furthermore, we examined the serum lipid profile of the obese mice. As shown in Fig. 5A, B and D, the TC, TG and LDL-c levels in the mice of HF groups were significantly increased compared with those of the Chow group mice (*p*<0.01). FME significantly lowered the serum TC, TG and LDL-c levels of HF+FME group compared to those of the HF group mice (*p*<0.05). However, no difference of the HDL-c level between HF and HF+FME groups was observed (Fig. 5C). Taken together, these results suggest that FME could alleviate the dyslipidemia in high-fat diet-induced obese mice.

### FME Improves Lipid Accumulation in Liver of C57BL/6 Mice

**Figure 6:**
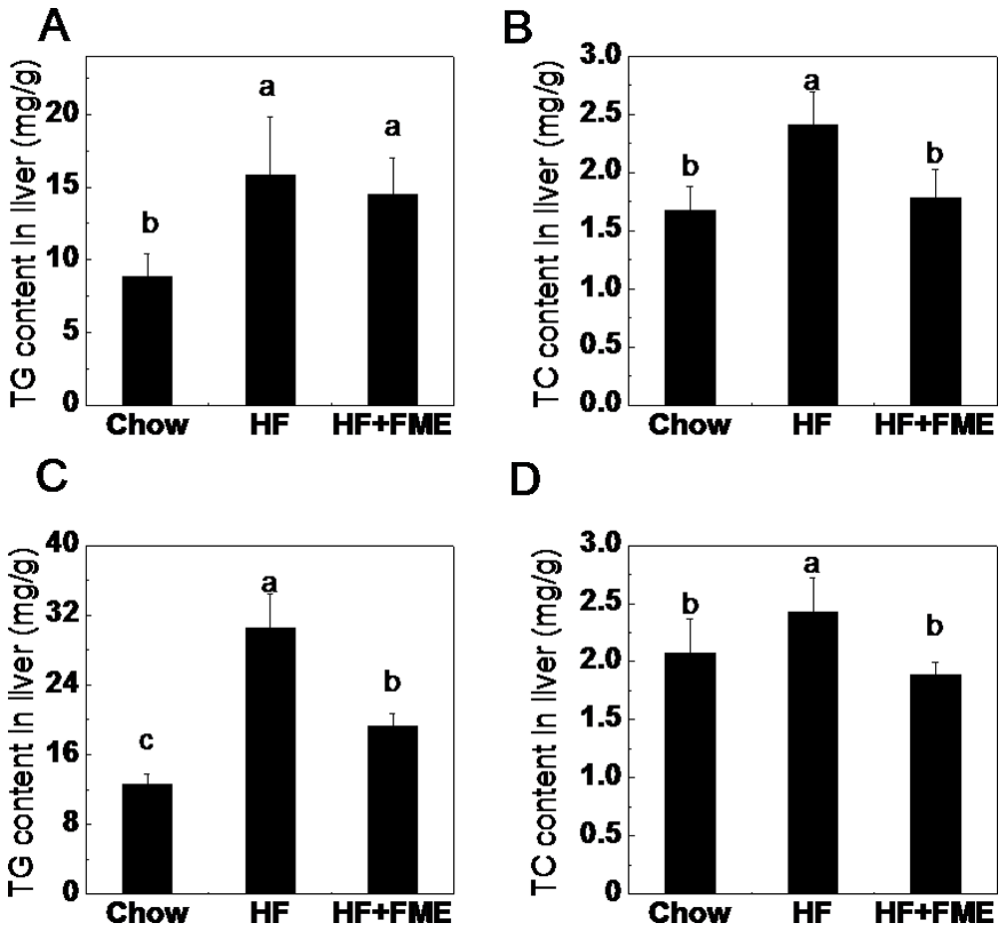
FME improves lipid accumulation in the liver of C57BL/6 mice induced by high-fat diet. (A): Triglyceride contents in the liver of preventive treatment. (B): Total cholesterol contents in the liver of preventive treatment. (C): Triglyceride contents in the liver of therapeutic treatment. (D): Total cholesterol contents in the liver of therapeutic treatment. Values are presented as mean ± SEM (n = 7). Values with different letters were significantly different (*p*<0.05).

Liver is one of the insulin-sensitive tissues and plays an important role in the processes of hyperglycemia and dyslipidemia. Therefore, we measured the TG and TC contents of liver tissues both in the preventive and therapeutic experiments. In the preventive experiments, the TG and TC contents were significantly increased in the HF diet-fed mice when compared to those in Chow group mice (Fig. 6A, B, *p*<0.01). FME treatment significantly decreased the TC accumulation in the liver of HF diet-fed mice (Fig. 6B, *p*<0.05). However, no significant difference in TG content of liver between HF and HF+FME groups was found (Fig. 6A). In the therapeutic experiment, the TG and TC contents in the liver of obese mice were also significantly higher than those in the control mice group, in the same way, FME treatment significantly lowered both TG and TC contents in the obese mice liver (Fig. 6C, D, *p*<0.05). These results suggest that FME attenuate the lipid accumulation in HF diet-fed mice liver.

### FME Influences the Expression of PPARα and its Target Genes *in vivo*

**Figure 7:**
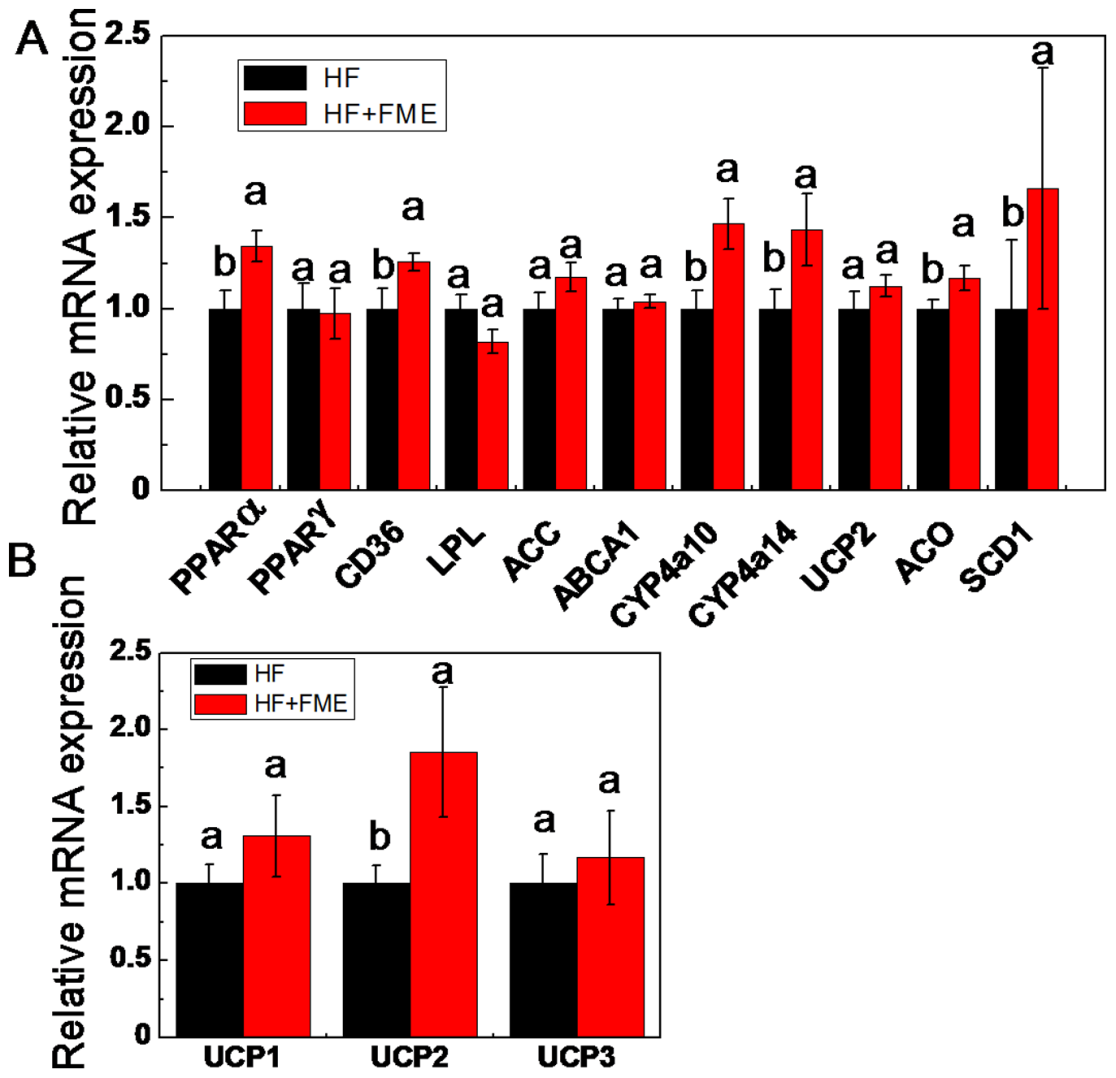
FME influences the expression of PPARα and its target genes in vivo. (A): The relative gene expression levels of PPARα and its target genes in the liver of FME-treated and HF-fed mice in the therapeutic treatment. β-actin was used as an internal control. (B): The relative gene expression levels of UCP1, UCP2 and UCP3 in the brown adipose tissue of FME-treated and HF-fed mice in the therapeutic treatment. Values are presented as mean ± SEM (n = 6). Values with different letters were significantly different (*p*<0.05).

To explore the mechanism of FME improving the metabolic disorders in high-fat diet-fed mice, we analyzed the gene expressions related to the glucose and lipid metabolism of liver. As shown in Fig. 7A, the mRNA level of PPARα was significantly increased in HF+FME mice group compared with that of the HF group. In addition, the mRNA levels of PPARα target genes such as cluster of differentiation 36 (CD36), cytochrome P450 4a10 (CYP4a10), cytochrome P450 4a14 (CYP4a14), acyl-CoA oxidase (ACO) and stearoyl-CoA desaturase-1 (SCD1) were also significantly increased in HF+FME group compared to those in HF group. However, the expression levels of lipoprotein lipase (LPL), uncoupling protein (UCP-2), acetyl coenzyme A carboxylase (ACC) and ATP-binding cassette transporter 1 (ABCA1) were not significantly influenced by FME treatment. In addition, the expression of PPARγ in the liver of experimental mice was also analyzed, but no significant difference between HF and HF+FME groups was observed. Taken together, these results suggest that FME may improve the metabolic disorders of obese mice partly though PPARα signaling.

Finally, we analyzed the expression of genes in brown adipose tissue (BAT), because BAT has been reported to play an important role in energy metabolism of mice. As shown in Fig. 7B, the expression of UCP-2 was significantly increased in BAT of HF+FME mice compared with that of HF group. However, no significant difference of the mRNA expression levels of UCP1 and UCP3 genes was observed between HF and HF+FME groups.

## Discussion

This study is aimed at investigating the effect of FME on the metabolic disorders in experimental mice. The results obtained showed that FME prevented the body weight gain and controlled the glucose and lipid levels in the C57BL/6 mice fed with high-fat diet.

Citrus fruit peels rich in flavonoids have been used as pharmaceutical component for hyperglycemia, hyperlipidemia and obesity for hundreds of years [12], [13], [32]. However, citrus peels are not eaten directly because of their bad taste, therefore, it is inadvisable to use citrus peels directly as dietary supplement. Distinguished from citrus, the whole FM fruit is usually consumed [27]. Although FM is rich in bioactive compounds which have strong antioxidant activity and has been a popular fruit in the south of China, little experiments had been carried out regarding its effects on metabolic disorders. In this study, the effects of FME on metabolic disorders in HF-diet induced obese C57BL/6 mice were investigated for the first time. And the results indicated that FME may be a potential candidate for diet supplement of metabolic disorders.

Citrus fruit extracts contain rich flavonoids [33]. In this study, we found that the major compounds of FME were neoeriocitrin and poncirin, of which the highest was neoeriocitrin. This is different from the reports about citrus fruits extracts, which showed that the major flavonoids are naringin, hesperidin and neohesperidin [34], [35]. This result is consistent with the report that the phenolic composition of the genus *Fortunella* is different from that of the genus *Citrus*, even though the two genera are closely related in taxonomy [36]. Neoeriocitrin has higher antioxidant activity than the similar compounds in citrus and has been studied for its effects on proliferation and osteogenic differentiation in osteoblast-like cell line (MC3T3-E1) [37], [38]. Poncirin has been reported to have many activities including anti-bacteria, anti-inflammation and inhibiting adipocyte differentiation [39]. Although the antioxidant and anti-inflammatory activities of neoeriocitrin and poncirin have been studied by many researchers, the research on their effects on metabolic disorders is rare. In the present study, our data indicated that FME attenuated not only the lipid and glucose levels in high-fat diet fed mice but also have therapeutic effects on metabolic disorders in obese mice, and these functions of FME could be attributed to neoeriocitrin and/or poncirin.

In our study, compared with the Chow group, the mice in HF group were induced to be obese by high-fat diet both in the preventive treatment and in the therapeutic treatment and we provided evidence that FME prevented obesity and obesity-related metabolic disorders. Fasting glucose levels in the preventive treatment, serum TG and LDL-c levels both in the preventive and therapeutic treatment were decreased to the normal (Chow) level by FME treatment. In the therapeutic experiment, glucose tolerance and insulin tolerance were significantly improved by FME treatment. Unexpectedly, however, the same effects were not observed in the preventive experiment. This discrepancy may be result from the shorter inducement of high-fat diet in the preventive treatment or other unknown reasons. Anyway, however, the fasting glucose levels of mice both in the preventive and therapeutic experiments were significantly decreased by FME supplement.

PPARs are ligand-activated transcription factors including PPARα, PPARβ and PPARγ belonging to the nuclear receptor family. PPARs are known to control the gene expression involved in lipid and glucose metabolism [40]. PPARα plays a pivotal role in the transcriptional regulation of genes involved in fatty acid uptake and oxidation [41]. Previous studies have shown that oleoylethanolamide decreased lipid content in hepatocytes as well as serum TC and TG levels in Zucker rats by upregulating the expression of PPARα in liver [42]. Cho also reported that naringenin treatment significantly lowered TG and TC in plasma and liver with increasing the PPARα expression in rats liver [43]. The target genes regulated by PPARα and involved in lipid metabolism and obesity include CD36, LPL, ACC, ABCA1, CYP4a10, CYP4a14, UCP2, ACO and SCD1 [44], [45]. CD36 is involved in fatty acid uptake, LPL is related with the hydrolysis of plasma triglycerides, ACC is participated in fatty acid synthesis, and ABCA1 is related to HDL metabolism [44]. CYP4a10 and CYP4a14 belong to the cytochrome P450 family which is involved in synthesis of cholesterol, steroids and other lipids [46]. UCP2 is a critical gene for fatty acid oxidation, ACO and SCD-1 are key genes in fatty acid metabolism [47], [48]. Our results showed that FME increased the expression of PPARα and its target genes including CD36, CYP4a10, CYP4a14, ACO and SCD1 in the liver of obese mice (Fig. 7A). In addition, the expression of UCP2 in BAT was also increased by FME in high-fat induced obese mice group. BAT plays an important role in energy metabolism of mice, and UCP1, UCP2 and UCP3 have been identified as the mediators of thermogenesis [49]. As previous reported, PPARα can induce the expression of UCP2 both in the liver and adipose tissues [44], [50], [51]. In the present study, the UCP2 expression in BAT but not in liver was increased significantly. Since UCP2 is a critical regulator of cellular glucose and lipid metabolism [52], we propose that FME may ameliorate metabolic disorders of obese mice partly though the regulation of PPARα signaling pathway.

In conclusion, our study demonstrated for the first time that FME had ameliorating effects on hyperglycemia, hyperlipidemia and hepatic lipid accumulation in high-fat induced obese mice. The expression levels of PPARα and its target genes involved in glucose and lipid metabolism in mice liver were increased by FME treatment. Our data suggest that FME may be a promising dietary supplement for obesity and obesity-related metabolic disorders.
